# Auditory Mismatch Negativity Response in Institutionalized Children

**DOI:** 10.3389/fnhum.2019.00300

**Published:** 2019-09-25

**Authors:** Irina Ovchinnikova, Marina A. Zhukova, Anna Luchina, Maxim V. Petrov, Marina J. Vasilyeva, Elena L. Grigorenko

**Affiliations:** ^1^Laboratory of Translational Sciences of Human Development, Saint-Petersburg State University, Saint-Petersburg, Russia; ^2^Department of Psychology, University of Houston, Houston, TX, United States; ^3^Department of Higher Nervous Activity and Psychophysiology, Biological Faculty, Saint-Petersburg State University, Saint-Petersburg, Russia; ^4^Department of Molecular and Human Genetics, Baylor College of Medicine, Houston, TX, United States; ^5^Child Study Center and Haskins Laboratories, Yale University, New Haven, CT, United States

**Keywords:** institutionalization, psychosocial deprivation, language development, auditory discrimination, event-related potentials, mismatch negativity, MMN

## Abstract

The attunement of speech perception/discrimination to the properties of one’s native language is a crucial step in speech and language development at early ages. Studying these processes in young children with a history of institutionalization is of great interest, as being raised in institutional care (IC) may lead to lags in language development. The sample consisted of 82 children, split into two age groups. The younger age group (<12 months) included 17 children from the IC and 17 children from the biological-family-care (BFC) group. The older group (>12 months) consisted of 23 children from the IC group, and 25 children from the BFC group. A double-oddball paradigm with three consonant-vowel syllables was used, utilizing native (Russian) and foreign (Hindi) languages. A Mismatch Negativity (MMN) component was elicited within a 125–225 ms time window in the frontal-central electrode. Findings demonstrate the absence of MMN effect in the younger age group, regardless of the living environment. Children in the older group are sensitive to native deviants and do not differentiate foreign language contrasts. No significant differences were observed between the IC and BFC groups for children older than 12 months, indicating that children in the IC have typical phonological processing. The results show that the MMN effect is not registered in Russian speaking children before the age of 12 months, regardless of their living environment. At 20 months of age, institutionally reared children show no evidence of delays in phonetic development despite a limited experience of language.

## Introduction

Institutional care (IC) remains a common type of placement for children raised without biological families in a number of countries, including the Russian Federation. Detrimental effects of IC have been well documented for different developmental domains, including language development. Studies show that children with an IC history, as a group, demonstrate a lack of comprehensive utterances at the age of 30 months when exposed to severe deprivation, such as in Romanian orphanages (Windsor et al., 2007), poor sentence comprehension, working memory deficits (Desmarais et al., 2012), and lower academic performance (Vorria et al., 2014) when exposed to institutional settings of variable quality. Documented deficits in language development have been associated with the length of institutionalization (Loman et al., 2009), especially for the receptive language domain (Eigsti et al., 2011; Desmarais et al., 2012). It has been argued that observed language deficits might be caused by the alteration of neural structures in IC children due to chronic stress and psycho-social deprivation (Eigsti et al., 2011), as well as impoverished input, a limited quantity and quality of linguistic input, and disrupted child-caregiver interactions (Windsor et al., 2007). Language is learned *via* social interaction (Kuhl et al., 2003), which institutionally reared children might be deprived of due to lack of caregivers’ responsiveness, low stability of the environment, and limited amount of child-directed interactions (Muhamedrahimov et al., 2005, 2014). Therefore, the lack of social interactions can result in poorer phonetic discrimination skills in children raised in institutions.

Event-related potential (ERP) studies of institutionalized children in the Russian Federation have shown that children of 30 months and above show attenuated processing of semantic incongruities manifested in the atypical N400 component, compared to peers raised in biological families (Zhukova et al., 2015). It is argued that an atypical neural response to semantic incongruity may reflect underspecified lexical representations or altered functional connectivity in children raised in IC in Russia. Data acquired from adults who were raised in institutions in the Russian Federation suggest that detrimental effects of institutionalization can be traced to adulthood and are manifested in atypical N400 and N170 ERP components (Petrov et al., 2018; Kornilov et al., 2019). It has been shown that adults with a history of institutionalization display reduced neural sensitivity to violations of word expectancy. The results suggest that language is a vulnerable domain in adults with a history of institutionalization, the deficits in which are not explained by general developmental delays and point to the pivotal role of the early linguistic environment in the development of the neural networks involved in language processing. No study to the best of our knowledge has considered very early stages of language processing in children raised in institutions.

The ability to extract native phonological patterns is one of the key components of language development. Studies have demonstrated that infants have an increased general sensitivity, being able to successfully discriminate between sounds of native and non-native languages, gradually becoming attuned to native language and reaching “perceptual narrowing” by the age of 12 months. Perceptual narrowing is an adaptive mechanism that helps to filter out irrelevant linguistic input through perceptual bias (Lewkowicz and Ghazanfar, 2009; Maurer and Werker, 2014). Importantly, the timing of perceptual narrowing can be extended by a number of factors including gestational age (Peña et al., 2012), maternal mental health (Weikum et al., 2012), diet (Innis et al., 2001), and bilingualism (Burns et al., 2007).

Perceptual narrowing has been commonly studied using neuroimaging techniques, including event-related brain potentials (Cheour et al., 2000; Kuhl, 2004), such as the mismatch negativity (MMN) component (Näätänen, 2003). This component is elicited in response to violations of expectation (Winkler, 2007) and has been widely studied as a neural correlate of phonological discrimination in response to changes in auditory stimulation (Duncan et al., 2009). It plays a pivotal role in speech perception; smaller amplitudes of the MMN component are assumed to reflect poorer speech-sound representations, and as language skills improve, MMN to speech sound contrasts to that language are enhanced (Winkler et al., 1999; Wible et al., 2004). The MMN component can be elicited even in the absence of a participant’s attention (Rivera-Gaxiola et al., 2005), and therefore has been widely used in studies with pediatric samples. It has been shown to be sensitive to speech-language and reading difficulties, which are characterized by the altered amplitude of this component compared to typically developing peers (Baldeweg et al., 1999; Cheour et al., 2000; Friederici et al., 2002; Leppänen et al., 2012; Neuhoff et al., 2012; van Zuijen et al., 2013).

Given the impoverished characteristics of the linguistic environment of IC (Windsor et al., 2007; Scott et al., 2011), we hypothesize that children raised in orphanages might demonstrate atypical phonological processing manifested in the discrimination of non-native language patterns after the age of 12 months due to the lack of social interactions in psychosocially depriving environments of institutions.

## Participants

A total of 130 children were recruited for the study. However, a number of children (*n* = 22) were excluded according to strict exclusion/inclusion criteria: (1) inability to provide at least 180-30-30 trials to Standard after Standard and Deviant stimuli accordingly (*n* = 13); (2) presence of medically recorded hearing problems (*n* = 1); or (3) diagnosed neurological disorder or neurological symptoms such as epilepsy, brain ischemia, or prenatal brain injury (*n* = 7). One participant was excluded due to previous exposure to the Hindi language; all other participants were Russian native speakers with no previous exposure to the Hindi language.

We inspected the age distribution among the remaining 108 children and identified outliers who were older than 21 months. Due to the unequal distribution of older children in the IC and biological-family-care (BFC) groups, we excluded observations of children who were older than 21 months of age (*n* = 26). The final dataset included ERP data from 82 participants. They were split into two age groups according to the age of hypothesized perceptual narrowing (Rivera-Gaxiola et al., 2005; Maurer and Werker, 2014): the younger age group before 12 months and the older age group after the age of 12 months.

The younger age group included 17 children from the IC group (*M* = 10.5 months, *SD* = 1.18, 11 males) from four baby homes, and 17 children from the BFC group (*M* = 10.1 months, *SD* = 1.09, 12 males). The older group consisted of 23 children from the IC group (*M* = 17 months, *SD* = 2.26, 11 males), and 25 children from the BFC group (*M* = 16.9 m, *SD* = 2.25, 13 males). The groups did not differ significantly by age or sex distribution.

Written consent for participation was obtained from the children’s official representatives, baby home officials or biological parents. The study procedure was approved by the Institutional Review Board (Ethical Committee) of Saint Petersburg State University, Russia.

## Method

To elicit the MMN ERP component, we used a passive double oddball paradigm (Conboy and Kuhl, 2011). Stimuli were comprised of stop consonant-vowel syllables. We used the 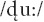 syllable as a standard stimulus, and /gu:/ and 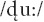 as the deviants. Standard /du:/ and deviant /gu:/ were classified as native language patterns; the deviant 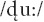 was classified as a foreign phonological pattern from Hindi. The experiment consisted of 1,500 trials, with 1,200 standard (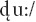) and 300 deviant (150 /gu:/ and 150 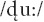) trials in total, therefore the ratio of standard to deviant syllables was 8:1:1 (Table 1).

**Table 1:** Types of auditory stimuli in the event-related potential (ERP) experiment.

Stimulus type	Syllable	Language, where the pattern is present	# of trials	Properties	Duration
Standard	/du:/	Russian and Hindi	1,200	Voiced dental	246 ms
Foreign deviant	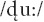	Hindi	150	Retroflex	242 ms
Native deviant	/gu:/	Russian and Hindi	150	Voiced velar	246 ms

Trials were split into three blocks with 500 stimuli each. Brief 5-min breaks were given between the trial blocks. The stimuli were recorded by a female native Hindi speaker using PRAAT audio software at a sample rate of 44,100 Hz, and presented at 70 dB (SPL) using a set of Yamaha NS-BP300 speakers. Stimuli were administered in a pseudo-randomized order to allow for at least three standard stimuli between deviants; the inter-stimulus interval was 600 ms.

## Procedure

The EEG signal was detected using a high-density EEG system via a PC laptop running PyCorder software (BrainProducts Inc.). Specifically, we used the actiCHamp amplifier (BrainProducts, Inc.) to record EEG from the scalp using 64 Ag/AgCl sintered active electrodes mounted in an elastic cap according to the standard montage using SuperVisc electrolyte gel. The signal was recorded using linked mastoids as the reference and digitized at 1,000 Hz.

Data of 31 participants were recorded with online filter settings of 0.10–30 Hz and data of 51 participants were obtained with online filter settings of 0.10–50 Hz. An additional notch filter at 50 Hz was applied to the data online. This inconsistency in data acquisition was attributed to a violation of the research protocol, which was handled at the preprocessing step.

All impedances were kept below 25 kΩ. During the recording, children sat on a caregiver’s lap and watched a muted cartoon on a laptop, while auditory stimuli were presented through open field speakers binaurally. Caregivers were instructed not to attend and/or react to stimuli. The EEG data were processed offline using BrainVision Analyzer software v 2.1 (BrainProducts Inc.). The signal was downsampled to 500 Hz. After visual inspection of the raw data for each participant, channels contaminated by noise were reconstructed using spherical spline interpolation. The signal was re-referenced to the common average reference. IIR filter (low cut-off: 0.10; high cut-off: 30 Hz) was applied to the signal in order to homogenize the filter settings across all participants, followed by a 50 Hz notch filter. We used Independent Component Analysis (ICA) to perform the ocular correction procedure. One of the frontal electrodes (FP1 or FP2 depending on the quality of the recoding) served as a blink marker channel for vertical activity. The difference between FP9 and FP10 electrodes served as a marker for horizontal activity. The Infomax algorithm was trained on a segment of data with a length of 140 s. The procedure was conducted in the semi-automatic mode. After the ICA matrix was computed, the ICA components were visually inspected for each participant with regard to their topographic location and relative impact on the data. The components that were contributing to blinks were set to zero. In total, a maximum number of five ICA components were set to zero for each participant.

After that data was segmented into epochs with 100 ms prestimulus (served as baseline) and 700 ms poststimulus intervals, semi-automatic artifact rejection was carried out. The criteria for artifact rejection were: a voltage step of no more than 50 μV in the segment; and an absolute voltage not exceeding ±110 μV in any of the EEG channels. Baseline correction was performed in relation to the prestimulus time mentioned above and local DC detrending was applied to the extracted segments. The segments were averaged separately for the three experimental conditions: Standard, Native Deviant, Foreign Deviant. Trials in which a Standard stimulus directly followed a Native Deviant/Foreign Deviant were not used in the analysis. Participants were administered different numbers of trials, depending on their distress level and functional state, with minimum of 716 and maximum of 1,500 trials. During the artifact rejection procedure, trials containing exceeding amounts of noise were removed from the analysis (number of removed trials ranged from 77 to 480 segments for each participant, *M* = 281.03, 320 *SD* = 192.26). Therefore, on average 637.07 trials for Standard condition were retained (*min* = 421, *max* = 877, *SD* = 126.36); 107.03 Native deviants (*min* = 74, *max* = 148, *SD* = 21.27) and 106.47 Foreign Deviants trials (*min* = 72, *max* = 247, *SD* = 21.18) were left after the artifact rejection.

## Results

First, we conducted a *t*-test to ascertain whether the grand average waveforms of Deviant and Standard stimuli significantly differed from zero. All 64 channels were included in the grand average waveforms. There was a significant effect for all experimental conditions, suggesting that a comparison of electric brain activity in response to different experimental conditions is meaningful. To identify the best time window for the MMN analysis two difference waveforms were computed: Native Deviant—Standard, and Foreign Deviant—Standard. A *t*-test was conducted to compare whether the computed difference waveforms significantly differed from 0, suggesting the presence of MMN effect. For the difference waveform between the Native Deviant and the Standard, statistically significant effect was found in the time window of 125–225 ms after stimulus onset. No significant effect for the difference waveform of the Foreign Deviant and the Standard was observed. Since a significant difference between Native Deviant and Standard conditions was found in the time window of 125–225 ms after the stimulus onset, this latency range was selected as the time window for subsequent analysis.

MMN is a component that is observed in the fronto-central electrode sites (Duncan et al., 2009), therefore we first focused our analysis on Left fronto-central (F3, FC5, C3, CP5, F5, C5, CP3, FC3), Midline fronto-central (FC1, Fz, CP1, CP2, Cz, FC2, AF3, AFz, F1, FCz, C1, C2, CPz, F2, AF4), and Right fronto-central (CP6, C4, FC6, F4, C6, FC4, F6, CP4) electrode sites. The younger and older groups of children were analyzed separately to account for potential differences in phonological processing due to perceptual specialization that occurs after the age of 12 months (Kuhl, 2004). In the younger age group, there was no significant effect of electrode cluster in predicting average amplitude differences across experimental conditions (*F*_(2,288)_ = 1.75, *p* = 0.17, Cohen’s *f* = 0.11), however in the older age group a significant effect of electrode cluster was found (*F*_(2,414)_ = 14.09, *p* < 0.001, *f* = 0.26). To account for those differences and to keep subsequent statistical analysis consistent across the age groups we moved to individual electrode analysis. The average amplitude in the Fz electrode was selected as an outcome variable in line with previous research (Näätänen et al., 2004; Bishop, 2007).

We utilized a factorial ANOVA to compare the main effects of group (IC/BFC) and stimulus type (Standard, Native Deviant, Foreign Deviant), as well as an interaction effect between group and stimulus type, using the average amplitude of the frontal central electrode (Fz) as an outcome variable. We calculated the mean amplitude for each participant and type of stimulus separately. Statistical analysis was conducted in each age group separately. Tukey correction for multiple comparison was used to correct for the number of experimental conditions in the analysis. Alpha level was 0.05.

**Figure 1:**
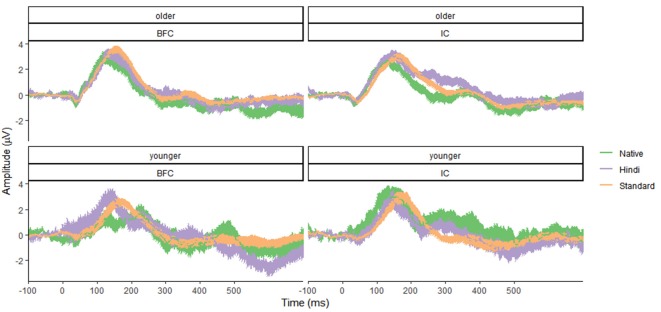
Mean amplitude characteristics of event-related potential (ERP) waveforms in the younger (bottom panel) and older (top panel) groups of participants—biological family (BFC, left panel) and children raised in institutional settings (IC, right panel) in response to three types of stimuli: Foreign Deviant, Native Deviant, and Standard. Shaded areas represent confidence intervals.

**Figure 2:**
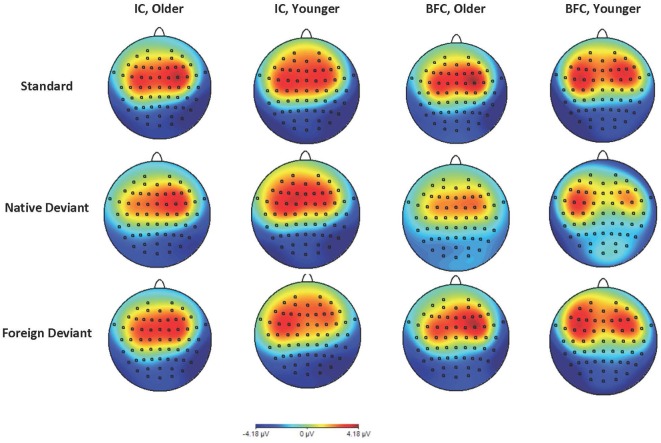
Topographic maps of ERP in the time window of 100 ms–200 ms in the younger and older groups of participants—biological family (BFC) and children raised in institutional settings (IC) in response to three types of stimuli: Foreign Deviant, Native Deviant, and Standard.

Results for the younger age group showed no significant effects of group (*F*_(1,96)_ = 1.37, *p* = 0.24, *f* = 0.12), stimulus type (*F*_(2,96)_ = 0.82, *p* = 0.45, *f* = 0.13), or their interaction (*F*_(2,96)_ = 0.73, *p* = 0.49, *f* = 0.12), suggesting that no MMN effect was registered. The group effect was not significant, indicating the absence of any significant differences between the IC and BFC groups in response to the auditory stimuli in the younger age group. Results for the older age group demonstrated that the type of stimulus effect was significant (*F*_(2,138)_ = 3.695, *p* = 0.027, *f* = 0.23), with greater negativity in response to the Native Deviant stimuli compared to the Standard stimuli [*M* = −1.02, *p* = 0.04, 95% CI (−2.02, −0.03)]. No significant differences were found between the Standard and Foreign Deviants (*p* = 0.977), as well as between the Native and Foreign Deviants (*p* = 0.067). The group effect was not significant for the older age group as well (*f* = 0.05), indicating the absence of any significant differences in phonological processing between the IC and BFC (Figures 1, 2; Supplementary Tables S1, S2). Also, there was no interaction effect between stimulus type and group factor (*f* = 0.04).

A *post hoc* power analysis revealed that we had 64% power to detect an effect size of *f* = 0.2 in the older age group, given the sample size of 48 children. In the younger age group (*n* = 34) we had 60% power to detect an effect of *f* = 0.2. We believe that the modest sample sizes in each group may have played a role in our inability to detect the significance of the statistical comparisons conducted, in particular in the younger age group.

## Discussion

Our results demonstrate the absence of any MMN effect in the younger age group from our sample, which contradicts the findings presented in the literature. Previously it has been shown that children before the age of 12 months have sensitivity to native, as well as foreign phonological patterns (Maurer and Werker, 2014), therefore we expected to see an MMN effect in the younger age group for the Native Deviant and Foreign Deviant stimuli. The absence of any MMN effect in the younger age group can be explained by the heterogeneity of this component in pediatric samples. It has been shown that the amplitude and polarity of the MMN component changes as a function of age (Friederici et al., 2002; Kushnerenko et al., 2002). Since in this analysis we have used average amplitude of electrical brain activity as an outcome variable, we hypothesize that the MMN effect could be attenuated due to averaging. Also considering the modest sample size, the study could be underpowered for detecting significant results.

In line with our prediction, our findings show that children in the older group are sensitive to native deviants and do not differentiate foreign language contrasts. These findings are in correspondence with the existing literature, which describes perceptual narrowing and reduced sensitivity to non-native language contrasts in typically developing children after the age of 12 months (Werker and Tees, 1984; Cheour et al., 1998; Rivera-Gaxiola et al., 2005). Specifically, Cheour et al. (1998) reported that infants at 6 months showed a discriminatory response to both native and non-native vowel stimuli, but that by the age of 12 months neural responses to the non-native vowel contrasts were attenuated. A study that also used Hindi non-native deviant consonants showed that children at 7 months of age reveal discrimination of both native and non-native phonetic contrasts, and lose sensitivity to non-native contrasts by the age of 11 months (Rivera-Gaxiola et al., 2005). In addition, we have replicated previous findings that suggest that the MMN effect is observed for native but not foreign language contrasts in typically developing children.

Contrary to our prediction, there was no significant group effect of institutional vs. family environment, indicating that children in the IC group, similar to typically developing peers in biological families, are not sensitive to foreign language contrasts without prolonged exposure to the foreign language. Our initial hypothesis posited that given the impoverished linguistic input in baby homes, children in IC would demonstrate sensitivity to foreign language patterns after the age of 12 months, revealing poorer phonetic representations and discrimination skills. This hypothesis was rejected as the data indicate the presence of significant stimulus type effect for native but not foreign deviants in the older age group compared to the standard stimulus for all children, regardless of their living environment. This study was one of the first attempts to investigate the neural processes underlying the language development of children in institutions using ERP.

Previous studies have demonstrated that children raised in IC demonstrate poor sentence comprehension (Desmarais et al., 2012), low scores in the expressive language domain coupled with hypoactivation of the Broca area (Helder et al., 2014), as well as structural changes and white matter abnormalities in brain areas associated with language, such as the left superior longitudinal fasciculus (Govindan et al., 2010) and arcuate fasciculus (Kumar et al., 2014).

Our study aimed to extend the existing literature by providing data on an intermediate language phenotype in IC children. We aimed to analyze preattentive lower-level language processing characteristics, thus choosing MMN as the component of interest. Our study suggests that the discriminability of auditory information is intact in children raised in institutions, opening up questions regarding the higher-order mechanisms that might explain language deficits in IC children. Thus, based on recent theoretical views of perception narrowing in general and the MMN component in particular as stages in the formation of prediction (and prediction error) in language processing (Bornkessel-Schlesewsky and Schlesewsky, 2019), it will be important to interrogate the IC-BFC group differences in other language-related negative ERP components (e.g., the LAN and N400).

The majority of studies on the MMN component published in Russia have used it as a marker of cognitive decline in various conditions, including stroke (Garin and Poverennova, 2008), dementia (Morozova et al., 2012), schizophrenia (Chepikova et al., 2015; Petrov et al., 2017), and exposure to radiation (Zhavoronkova et al., 2010), or as a method of studying attention in typically developing adults (Hodanovich et al., 2009; Gorjainova et al., 2019). Research on Russian children using the MMN component is more scarce. It has been used to study cognitive functions in infants (Vasil’eva et al., 2015) and brain development in children raised in the harsh climatic conditions of the Russian North (Nagornova et al., 2018); also MMN has been proven to be an effective measure for identifying attentional deficits. Moreover, a study using the MMN component established auditory processing deficits in children with motor dysphasia (Savel’eva et al., 2015). No studies published in Russia have considered MMN characteristics in children younger than 3 years of age or children raised in impoverished environments, making this study the first of its kind.

The current study had a number of limitations. First, given the heterogeneity of the MMN component (in terms of spatial distribution and amplitude polarity across developmental milestones; Bishop, 2007), the current sample size might not have been large enough to yield adequate statistical power. Second, the Foreign deviant stimuli were shorter in duration compared to the Standard and Native deviants. These durations should be considered in designing future studies, however, this aspect is unlikely to affect the results, as we observed no significant differences in the responses to Foreign deviants compared to Standard stimuli. Third, it has been reported that MMN amplitude is related to the amount of speech exposure (Marklund et al., 2019); thus, it is important to explore the specifics of language interaction in the IC group (e.g., the amount of received and produced speech by a child), which, to our knowledge, has never been done. Finally, there are multiple MMN paradigms—e.g., whole word storage MMN, syntactic MMN (Hanna et al., 2017)—we utilized only one, which limits the generalizability of our conclusions. Finally, the auditory stimuli were presented through open field speakers, and caregivers were not wearing sound-canceling headphones. Even though they were instructed not to attend to stimuli, the study does not control for potential caregiver’s impact on child attention to the stimuli.

Future studies should continue to interrogate the mechanics of the observed language deficits in individuals who have experienced early institutionalization by extending the MMN paradigm to include other types of stimuli and exploring neurobiological components related to higher-level language processing. In this way, potential biomarkers of language problems in the subpopulation of institutionalized children may be identified.

## Data Availability

The datasets generated for this study are available on request to the corresponding author.

## Ethics Statement

Written informed consent for participation was obtained from the children’s official representatives, baby home officials or parents. The study procedure was approved by the Institutional Review Board (Ethical Committee) of Saint Petersburg State University, Russia.

## Author Contributions

EG led the development and conceptualization of the overall research effort. MV contributed to the study and stimuli design. IO, MZ, AL, and MV collected the data. IO, MZ, AL, and MP preprocessed the EEG data and performed the statistical data analysis. IO, MZ, and EG drafted the first version of the manuscript. IO and MZ prepared the figures.

## Conflict of Interest Statement

The authors declare that the research was conducted in the absence of any commercial or financial relationships that could be construed as a potential conflict of interest.
