# Supplementary material for: Auditory Mismatch Negativity Response in Institutionalized Children
Source: Front Hum Neurosci. 2019 Sep 25;13:300. doi: 10.3389/fnhum.2019.00300 (PMC6774417; doi:10.3389/fnhum.2019.00300)
Supplement: Supplementary file 1 [file Table_1.DOCX]

Supplementary Material

*Table 1.* ANOVA table with results for the older age group.

Type of stimuli: Foreign Deviant, Native Deviant, and Standard. Pooling: Left frontal, Midline frontal and Right frontal electrode sites. Group: biological family care and institutional care.

Outcome variable: mean amplitude
 Df Sum Sq Mean Sq F value Pr(>F)
Type.of.stimuli 2 46.65 23.326 9.5873 8.508e-05 ***
Pooling 2 68.54 34.272 14.0864 1.206e-06 ***
Group 1 3.52 3.520 1.4470 0.2297
Type.of.stimuli:Pooling 4 4.54 1.135 0.4664 0.7604
Type.of.stimuli:Group 2 1.38 0.692 0.2846 0.7525
Pooling:Group 2 1.59 0.795 0.3266 0.7216
Type.of.stimuli:Pooling:Group 4 3.46 0.865 0.3554 0.8403
Residuals 414 1007.26 2.433

*Table 2.* ANOVA table with results for the younger age group.

Type of stimuli: Foreign Deviant, Native Deviant, and Standard. Pooling: Left frontal, Midline frontal and Right frontal electrode sites. Group: biological family care and institutional care.

Outcome variable: mean amplitude
 Df Sum Sq Mean Sq F value Pr(>F)
Type.of.stimuli 2 34.65 17.3232 7.0646 0.001011 **
Pooling 2 8.59 4.2963 1.7521 0.175252
Group 1 0.02 0.0208 0.0085 0.926695
Type.of.stimuli:Pooling 4 6.87 1.7176 0.7005 0.592180
Type.of.stimuli:Group 2 11.50 5.7513 2.3454 0.097633 .
Pooling:Group 2 6.73 3.3673 1.3732 0.254936
Type.of.stimuli:Pooling:Group 4 0.18 0.0441 0.0180 0.999365
Residuals 288 706.20 2.4521

Signif. codes: '***'< 0.001; '**' < 0.01; '.' < 0.1
